# Dietary Ruminant Enteric Methane Mitigation Strategies: Current Findings, Potential Risks and Applicability

**DOI:** 10.3390/ani13162586

**Published:** 2023-08-10

**Authors:** Tomas Lileikis, Rasa Nainienė, Saulius Bliznikas, Virginijus Uchockis

**Affiliations:** 1 Department of Animal Nutrition and Feedstuffs, Animal Science Institute, Lithuanian University of Health Sciences, R. Žebenkos 12, 82317 Baisogala, Lithuania; virginijus.uchockis@lsmu.lt; 2 Department of Animal Breeding and Reproduction, Animal Science Institute, Lithuanian University of Health Sciences, R. Žebenkos 12, 82317 Baisogala, Lithuania; rasa.nainiene@lsmu.lt; 3 Analytical Laboratory, Animal Science Institute, Lithuanian University of Health Sciences, R. Žebenkos 12, 82317 Baisogala, Lithuania; saulius.bliznikas@lsmu.lt

**Keywords:** methane, emission, mitigation, ruminant, feed and forages

## Abstract

**Simple Summary:**

This article aims to explore various ruminant enteric methane mitigation strategies and delve into their underlying modes of action. Furthermore, it addresses the importance of ruminant enteric methane mitigation in the context of climate change and its potential impact on global warming. The article also highlights the need for interdisciplinary research and collaboration to develop comprehensive and practical solutions. By considering the ecological, economic, and social implications of these strategies, policymakers and stakeholders can make informed decisions to reduce methane emissions while ensuring the sustainability of livestock production systems.

**Abstract:**

This review examines the current state of knowledge regarding the effectiveness of different dietary ruminant enteric methane mitigation strategies and their modes of action together with the issues discussed regarding the potential harms/risks and applicability of such strategies. By investigating these strategies, we can enhance our understanding of the mechanisms by which they influence methane production and identify promising approaches for sustainable mitigation of methane emissions. Out of all nutritional strategies, the use of 3-nitrooxypropanol, red seaweed, tannins, saponins, essential oils, nitrates, and sulfates demonstrates the potential to reduce emissions and receives a lot of attention from the scientific community. The use of certain additives as pure compounds is challenging under certain conditions, such as pasture-based systems, so the potential use of forages with sufficient amounts of plant secondary metabolites is also explored. Additionally, improved forage quality (maturity and nutrient composition) might help to further reduce emissions. Red seaweed, although proven to be very effective in reducing emissions, raises some questions regarding the volatility of the main active compound, bromoform, and challenges regarding the cultivation of the seaweed. Other relatively new methods of mitigation, such as the use of cyanogenic glycosides, are also discussed in this article. Together with nitrates, cyanogenic glycosides pose serious risks to animal health, but research has proven their efficacy and safety when control measures are taken. Furthermore, the risks of nitrate use can be minimized by using probiotics. Some of the discussed strategies, namely monensin or halogenated hydrocarbons (as pure compounds), demonstrate efficacy but are unlikely to be implemented widely because of legal restrictions.

## 1. Introduction

The human population is expected to reach 9.7 billion by 2050 and this growth will result in increased pressure on the global food chain [1]. The livestock sector plays a crucial role in global food production and sustains the livelihoods of billions of people worldwide. Among the various livestock species, ruminants, including cattle, buffaloes, sheep, and goats, are particularly significant due to their unique digestive system, which enables them to convert fibrous plant materials into high-quality food sources. However, the enteric fermentation process in ruminants gives rise to a potent greenhouse gas (GHG), methane (CH_4_), which significantly contributes to global warming.

Reducing enteric methane emissions from ruminants has become a major concern in recent years due to its environmental impact and role in climate change. Concentrations of CH_4_ in the atmosphere have shown an increase of 150% since 1750 [2]. The livestock sector is responsible for 5% of total anthropogenic GHG emissions, and enteric fermentation is the major source of the sector’s CH_4_ emissions constituting 39.1% of the total [3]. The lifetime of methane in the atmosphere is 12.4 years, during which methane is eventually oxidized to CO_2_ and is subsequently fixed by plants through photosynthesis. Despite the relatively short lifetime of methane, it has approximately 28 times more global warming potential than carbon dioxide (CO_2_) on a 100-year timescale and is 84 times more potent on a 20-year timescale [2]. It is imperative to develop effective and sustainable strategies to mitigate methane production in ruminants without compromising their productivity or welfare. In addition, methane formation accounts for significant energy losses—up to 12% of gross energy intake in ruminants is lost as CH_4_ [4]. Consequently, the mitigation of methane emissions could help to improve animal productivity.

## 2. Enteric Methane Emission Mitigation Strategies

### 2.1. Composition and Quality of Feeds

#### 2.1.1. Concentrate Feeding

Increasing the amount of concentrates in animal diets has been a widely used strategy for many years, although this shift was more influenced not by the efforts to reduce emissions, but by the efforts to increase livestock productivity. By increasing the amount of concentrates (especially those containing starch) in the diet, the number of cellulolytic bacteria decreases and the number of amylolytic bacteria increases due to the changing ratio of substrates [5,6]. Amylolytic bacteria produce propionate instead of acetate, thus changing the acetate/propionate ratio [7]. Due to the changed ratio of volatile fatty acids (VFA), the amount of hydrogen available to methanogenic archaea decreases, and the pH of the rumen drops, which further inhibits/reduces the populations of cellulolytic bacteria, protozoa, and methanogens [8,9]. Wang et al. [10] elucidated the shift to propionate formation from the perspective of hydrogen flow—an increase in available hydrogen in the rumen due to improved digestibility of nutrients thermodynamically favors propionate production. Furthermore, some of the rumen microbiota are sensitive to fluctuations in partial H_2_ pressure, when even a slight increase in H_2_ in the rumen negatively affects the degradation of plant material [11,12,13]. More concentrates in rations would also result in an increased digesta passage rate from the rumen, which directs more nutrients to intestinal digestion, thus facilitating additional reductions in methane emission [14,15,16]. Van Gastelen et al. [17] analyzed 24 studies where the forage/concentrate ratio was investigated as a CH_4_ reduction strategy and found that an average increase of 386 g/kg DM [9] in concentrates decreased CH_4_ intensity (g/kg of product) by 10% for sheep, 27% for dairy cattle, and 31% for beef cattle.

It is worth noting that increasing the amount of concentrates in the diet up to 30–40% decreases methane emissions relatively linearly, but when the amount of concentrates exceeds 80%, a sharp drop in methane production is observed [8,18,19]. However, rations with such a high percentage of concentrates increase the risk of subacute or acute acidosis, laminitis, liver abscesses, and other disorders [8,20]. In addition, when the amount of concentrates in rations exceeds 50–55%, a negative effect on the quality of milk is evident [8,21].

The use of concentrates can indirectly increase the total GHG emissions of farms as more intensive cultivation of cereal crops requires more herbicides, pesticides, and fertilizers, and the application of these substances requires heavy agricultural machinery, so the resulting upstream emissions could offset the GHG amount reduced through enteric fermentation while feeding such feedstuffs. One of the most important things worth mentioning is that by using diets with large amounts of concentrates, the physiological characteristics of the ruminant to convert indigestible fiber into high-quality protein sources available to humans, such as meat and milk, are not used as intensively, and due to climate change and the demographic explosion of the human population, the use of cereal crops will likely become more and more essential for the nutritional needs of mankind and not for ruminant feed [22,23].

#### 2.1.2. Forage Type and Quality

Although methane production g/kg of dry matter intake (DMI) is higher in animals fed higher forage/concentrate ratios, forage remains a promising method to reduce methane emissions since there is a significant variation in CH_4_ production between different types of forages used [22]. High-quality forages, such as young grass and legumes, contain lower amounts of cell wall components and higher amounts of protein and easily digestible carbohydrates, thus increasing DMI and reducing the mean retention time (MRT) of feed particles in the rumen [24]. Consequently, absolute CH_4_ production per animal might increase but a decrease in CH_4_ yield g/kg DMI is evident, as well as a decrease in methane intensity due to increased animal productivity [17,25]. As forages mature, amounts of neutral detergent fiber (NDF) and acid detergent fiber (ADF) increase, and such feeds become less palatable and harder to digest. Depending on different animal characteristics, such as species, age, breed, type, sex, and physiological state (maintenance, pregnancy, lactation), more mature forages might not be of sufficient nutritional value for high-producing animals and could increase CH_4_ production while decreasing animal productivity, thus elevating CH_4_ intensity [26]. While nutritional value is one of the most important traits of herbage, the newest research proves that it is not the only criterion that could be studied for the potential to mitigate methane emissions. Recently, da Cunha et al. [27] proved that sward structure and its interactions with the nutrient content of forage have better explanatory power in predicting DMI, average daily gain (ADG), and CH_4_ emissions than nutrient content alone. Apart from the nutritional approach described above, well-managed grazing systems could indirectly mitigate emissions through extensive carbon sequestration [3,28,29].

Secondary metabolites in plants (tannins, saponins, essential oils) have a toxic effect on bacteria, protozoa, and methanogenic archaea. Due to commensal relationships, changes in bacterial and protozoan populations also affect the populations of methanogenic archaea, consequently decreasing methane production [18,30].

Fundamentally, it is practically impossible to optimize ruminant diets in such a way that roughage can be entirely removed without causing health problems to the animal. Secondly, in different parts of the world, due to agroecological and other factors, forages in ruminant diets are dominated by different plant species, and there is a considerable variation in the production of enteric methane between different types of forages. Thirdly, in the USA, Canada, and most European countries, the animal-safe use potential of concentrates has already reached or is approaching its maximum, and forages are the main source of energy for ruminants in many developing countries due to their availability and cost [20]. For these reasons, and because of the potential for this strategy to be applied in different geographic areas, it is likely that adjustments in the quality and composition of forages will be a significant part of an integrated, multistage emission reduction plan [22].

#### 2.1.3. Feed Preservation

Different feed preservation technologies also affect CH_4_ emissions, but, to the author’s knowledge, the literature on this topic is limited. The intensity of methanogenesis tends to decrease when the feed is preserved, e.g., ensiled rather than dried. Such a trend can be explained by the fact that ensiled feeds are already fermented to a certain level, thus becoming easier and faster to digest [18,31,32].

To reduce methane emissions with the help of roughages, it would be worthwhile to pay attention to improving the quality of forage and its availability by testing and applying different grazing strategies, choosing plant species with higher digestibility and nutritional value, and using early-cut forages for ensiling while enhancing the quality of the preservation process itself.

### 2.2. Synthetic Compounds

#### 2.2.1. 3-Nitrooxypropanol

3-nitrooxypropanol (3-NOP) is a synthetic organic compound that was first synthesized in 1990 and patented in 2012 as a potent methanogenesis inhibitor [33,34]. The molecular structure of 3-NOP resembles the structure of methylated coenzyme M (methyl-CoM) [35,36].

Coenzyme M, so named because of its function, participates in methanogenesis as a carrier of a methyl group and is the smallest known cofactor. Methyl-CoM is the last intermediate compound that completes all pathways of methanogenesis (hydrogenotrophic, acetoclastic, methylotrophic) [37]. The last reaction in the methanogenesis pathway is catalyzed by the enzyme methyl-CoM reductase (MCR). This enzyme splits the methyl group from methyl-CoM and is found in all methanogenic archaea [38,39]. MCR is also found in methanotrophic archaea which carry out a reverse process [40]. For MCR to be active, the nickel ion in the composition of the enzyme must be in a Ni(I) oxidation state [35]. Because of the structural similarity of 3-NOP to methyl-CoM, it binds to MCR and, by oxidizing the nickel ion, briefly deactivates the enzyme. It was established that 3-NOP can bind to MCR in two different ways—both nitrate ester and hydroxyl groups can be located at the distance of electron transfer from Ni(I) [35].

It was found that the effectiveness of 3-nitrooxypropanol decreases as the amount of NDF in the feed increases (for every 10 g/kg DM NDF, the effectiveness of 3-nitrooxypropanol decreases by 1.64 ± 0.33%) [41], while increasing the amount of 3-NOP in the feed exerts an increase in the anti-methanogenic effect by 2.56 ± 0.55 percent for every 10 mg/kg DM of 3-NOP added. Therefore, when calculating the supplement dose, the dietary composition of animal feed should be considered (especially NDF) [42]. Due to the influence of NDF on the inhibitory properties of 3-NOP, a higher efficiency of this compound can be expected in intensive farming systems due to the higher amount of concentrates dominating animal diets.

Vyas et al. [43] indicated methane emission reduction during the study conducted with beef cattle, in which animals were fed backgrounding (70% of the ration consisting of barley silage) and finishing (87% of the ration consisting of barley grain) rations. The doses of 3-nitrooxypropanol used were 100 mg/kg DM (low dose) and 200 mg/kg DM (high dose). The observed methane emission reductions while feeding a backgrounding diet were 17% and 34% for low and high doses, respectively. For the finishing diet, the emission reduction was much more pronounced in the high 3-NOP dose group, resulting in an 84% decrease, while only a 12% decrease was observed in the low-dose group. McGinn et al. [44] also noted a large reduction of 70% in methane production in beef cattle fed a high-concentrate finishing diet at 125 mg 3-NOP/kg DM. Arndt et al. [23] in a published meta-analysis noted that in eleven studies conducted with 3-NOP, daily reduction of CH_4_ emissions ranged from 29% to 47%.

Research conducted with dairy cattle suggests that smaller amounts of 3-NOP are enough to achieve substantial results in mitigating CH_4_ emissions from cattle of this type. Van Gastelen et al. [45] noted that 60 mg/kg DM was enough to reduce CH_4_ production (g/d) by 28.2%, 37%, and 38% in animals fed a grass-silage-based diet, a grass silage and corn silage mixed diet, and a corn-silage-based diet, respectively. An amount of 80 mg/kg DM of 3-NOP was even more effective, reducing methane production by 31.4%, 42%, and 45.1% when feeding the aforementioned rations, respectively. The same 3-NOP dose of 60 mg/kg DM was used in the trial by Melgar et al. [46], resulting in a 26% reduction in daily methane emissions.

The literature on the effect of 3-NOP on methanogenesis in sheep is very scarce with, to the author’s knowledge, only one publication on the aforementioned topic. Martinez-Fernandez et al. [47] reported a 25% decrease in methane production per kilogram of DMI at 14 d of treatment and the reduction persisted at 30 d at 21%. While one could expect similar results replicated with sheep as with beef or dairy cattle because of the particular mode of action of 3-NOP, there remain several important yet undefined areas regarding sheep trials, such as the effects of 3-NOP on physiological and physiochemical parameters in sheep, growth performance, quality of production, etc.

While there is some evidence suggesting that increasing amounts of 3-NOP negatively affect DMI, the data are inconsistent. For example, Alemu et al. [48], Vyas et al. [43,49], and Kim et al. [50] reported trends/statistically significant decreases in DMI for beef cattle when supplemented with 3-NOP. On the contrary, in their other studies, Kim et al. [51] and Alemu et al. [52] found no effect of 3-NOP on DMI. A meta-analysis of 12 studies carried out by Jayanegara et al. [53], which included beef cattle, dairy cattle, and sheep, also found no negative effect of 3-NOP on DMI. Interestingly, a reduction in DMI during trials was observed in the studies with beef cattle, but not with dairy cattle. As to why that is, it is still under debate. A number of studies agree with the idea proposed by Allen et al. [54] that increased proportions of propionate and subsequent oxidation in the liver induces satiety. Other researchers proposed different ideas in their studies. These include the difference between the trials themselves (short-term vs. long-term, etc.) [51], variation in dosage of 3-NOP (smaller doses used for dairy cattle), or diet composition (high-forage, high-concentrate, etc.) [41,50,51,52,55].

Although the literature indicates efficiency variations between different groups of ruminants (sheep, dairy cattle, beef cattle), 3-NOP, due to its specific and selective mechanism of action (participating in the last step of methanogenesis), can be useful for reducing the amount of CH_4_ released by different ruminant groups and species [35]. The effectiveness of all compounds used to reduce CH_4_ emissions is also influenced not only by the species of the animal but also by the composition of its diet, the dose of the compound or substance, and the method and frequency of administration, and 3-NOP is no exception.

#### 2.2.2. Ionophores

Ionophores are a class of antimicrobial agents with bacteriostatic and coccidiostatic effects. These substances interfere with the intracellular and transmembrane ion movement of some bacteria and protozoa found in the digestive tract. The most widely used substance of this class in animal husbandry is monensin, which was first isolated in 1967 from *Streptomyces cinnamonensis* [56].

Monensin in Europe is approved only for use in dairy cattle as a slow-release capsule for the prevention of ketosis and is used as a coccidiostat in the poultry industry. In the USA, this ionophore is widely used in ruminant feeding as a substance that improves ruminant energy metabolism and increases the efficiency of consumed feed. The effect of monensin on methanogenesis is indirect:

1. The mechanism of action of this antimicrobial provides a competitive advantage to propionate-producing bacteria that use hydrogen for propionic acid synthesis, thus competing with methanogenic archaea for it [57].
2. Monensin affects protozoa, which are the main producers of hydrogen in the rumen [58].

The results of the studies with monensin vary widely in the literature. Appuhamy et al. [59] in their meta-analysis found that a dose of 32 mg/kg DMI monensin administered to beef cattle reduced CH_4_ production by an average of 19 g/d, while the results on dairy cattle were marginal (6 g/d). Such differences were speculated to arise from the fact that the DMI of dairy cattle was almost three times higher than that of beef cattle (18.6 vs. 7.2 kg/d) while the doses of monensin used were larger in beef cattle than in dairy cattle (32 vs. 21 mg/kg of DMI).

Benchaar [60] reported that a monensin dose of 24 mg/kg of DMI in dairy cattle had no effect on DM consumption, feed digestibility, and CH_4_ production. Almeida et al. [61], after analyzing 10 trials where ionophores were used (mainly monensin), found that the average reduction in methane yield achieved was only 4%. Melchior et al. [58] used monensin at a dose of 150 mg/head/day and also observed no effect on methane production. Grainger et al. [62] used a high dose (471 mg/head/day) of monensin in their study with dairy cattle but also found no reduction in CH_4_ emission.

There is very limited evidence in the literature regarding the effects of monensin on methanogenesis in sheep. Zhang et al. [63] found that a 40 mg/kg of DM dose of monensin suppressed CH_4_ production in female lambs by 12.7%. However, more research on this topic is needed to elucidate the effects of monensin feeding in sheep production systems.

It could be hypothesized that due to the improved energy metabolism and feed efficiency, animals raised for meat could reach the target weight faster because of a possible increase in ADG [64], so the total methane emission released during the entire life of an animal could be lower, but due to the extremely different data obtained from the studies conducted with monensin, the use of this ionophore to reduce the amount of methane is a questionable strategy. Furthermore, due to legal restrictions in the European Union and some countries of the world and the growing pressure to reduce the use of antimicrobials in the livestock sector, the use of monensin to reduce methane emissions does not seem to be a sustainable long-term solution to the problem [8].

#### 2.2.3. Halogenated Hydrocarbons

Halogenated C_1_ hydrocarbons (HHC), also called halogenated methane analogs, are organic compounds composed of carbon and hydrogen atoms, with one or more halogen element (fluorine, chlorine, bromine, or iodine) covalently bonded to the carbon backbone. This group includes such compounds as bromoform, chloroform (CF), and bromochloromethane (BCM). Due to the high redox potential of these compounds and their structural similarity to some intermediate products of methanogenesis, it is believed that they affect this process both directly and indirectly as elucidated by Yu et al. [65]. The direct inhibitory effect of these substances is based on the fact that they bind to corrinoid and porphinoid enzymes in the cell, which, due to the metal ions (e.g., cobalt, nickel) in their composition, have a strong attraction for substances with high redox potential. These enzymes, combined with halogenated hydrocarbons, catalyze dehalogenation and thus divert the flow of electrons away from methanogenesis. In addition, the hydrocarbons attached to the enzymes prevent the normal substrate (methyl group) from attaching to them. After the dehalogenation stops, enzymes containing corrinoids and porphinoids are released and continue to participate in the process of methane formation. The indirect effect of halogenated hydrocarbons is thought to be exerted by binding to protein-bound corrinoids and porphinoids. When the archaeal cell lacks these proteins needed for methanogenesis enzymes, the process slows down. In addition, the intermediate products of dehalogenation compounds direct the electron flow away from the methanogenesis steps [65].

Knight et al. [66] during their trial administered 1.5 mL CF to non-lactating dairy cattle. By day 4–5 of the study, a strong decrease in methane production was observed, but thereafter methane release slowly increased until it reached 62% of pre-treatment methane emissions at the end of the trial. Tomkins et al. [67] during a trial with beef cattle used BCM at a dose of 0.3 g/100 kg live weight (LW) twice daily and observed a 60% and 50% reduction in methane production on days 30 and 90 compared to the control group. Abecia et al. [68] also used BCM at a dose of 0.3 g/100 kg LW and reported a 33% decrease in methane emissions in dairy goats (Table 1). The authors did not detect any effect on the abundance of rumen bacteria and protozoa. The total archaeal abundance was also not affected, but a redistribution of different species of archaea was evident, thus reinforcing the idea that, in terms of methanogenic activity, a significant variation exists between the species of archaea.

The studies conducted with halogenated hydrocarbons show that archaeal populations in the rumen might become at least partially resistant to such compounds. In addition, a significant number of halogenated hydrocarbons (including BCM) deplete the ozone layer and are, therefore, banned or strictly controlled by the Montreal Protocol in many countries. Other compounds, e.g., chloroform, are recognized as toxic and carcinogenic, so their use in livestock production systems raises both ethical and public health issues. For all these reasons, as well as legal difficulties in registering such substances for commercial use, halogenated hydrocarbons as pure compounds alone are unlikely to be used to reduce methane emissions from ruminant livestock.

### 2.3. Plants and Their Bioactive Compounds

#### 2.3.1. Macroalgae

Marine macroalgae, commonly known as seaweeds, are receiving more and more attention from the scientific community as a possible enteric methane emission mitigation option. Macroalgae, depending on their color, belong to three main phyla: *Chlorophyta* (green), *Phaeophyta* (brown), and *Rhodophyta* (red). It is estimated that there are around 6200 different red macroalgae species and around 1800 species of brown and green macroalgae each, making red algae the most diverse [69]. Research on the topic of methane emission mitigation has been carried out using seaweed from all three phyla, but so far the most promising and widely studied are two species from the same genus of red seaweed—*Asparagopsis taxiformis* and *Asparagopsis armata*. Although partial mitigation of methane production can be attributed to various compounds (phlorotannins, saponins, alkaloids, flavonoids, etc.) [70] and interactions between them, the main molecule of interest in *A. taxiformis* and *A. armata* is the halogenated compound bromoform (CHBr_3_) [9,71,72]. The mode of action of bromoform is the same as that of other halogenated compounds, which is discussed in the other section of the article.

Roque et al. [73] in their trial with dairy cattle used freeze-dried *A. armata* at two different inclusion rates of 0.5% (low) and 1% (high) on an organic matter (OM) basis. Methane production (g/d) decreased by 26.4% and 67.2% for low- and high-inclusion groups, respectively. A 10.8% DMI reduction was observed in the low-inclusion group of *A. armata* with no significant changes in BW or milk composition. However, researchers observed negative effects on DMI (−38%), BW gain (9.72 kg less), and milk yield (−11.6%) in the high-inclusion group, compared to the control, while the adjusted feed conversion efficiency was significantly greater (0.95 kg milk/kg intake) in the high-inclusion group. Stefanoni et al. [74] used *A. taxiformis* at inclusion rates of 0.25% (low AT) and 0.5% (high AT) on a DM basis. The authors reported an average of 34.4% CH_4_ emission reduction in the high AT group, but no significant changes in the low AT group were observed, compared to the control. Interestingly, the reduction in CH_4_ production was observed only in periods 1 and 2 of the experiment (65% and 55% reduction, respectively), while no effect was observed in later periods 3 and 4. Also, a DMI reduction of 7.11% and a milk yield decrease of 6.5% were observed in the high AT group, along with a decrease in lactose yield. Alvarez-Hess et al. [75] examined the effect of *A. armata* steeped in canola oil with (ASP1) and without (ASP2) seaweed biomass removed. Doses of ASP1 and ASP2 were 136 and 145 g/d, respectively. The authors reported a decrease in methane production by 44% and 39% for the ASP1 and ASP2 groups, respectively. No significant effect on DMI or milk yield was observed.

In a study with beef cattle, Roque et al. [76] used freeze-dried *A. taxiformis* at inclusion rates of 0.25% (low) and 0.5% (high) on an OM basis. The authors reported a 45 and 68% reduction in methane yield for low- and high-inclusion groups, respectively. There was a tendency for DMI to decrease (8%) in the low-inclusion group and a statistically significant decrease of 14% in the high-inclusion group with no significant effect on ADG or carcass quality observed. Consequently, the researchers observed a tendency for feed conversion efficiency (FCE) to increase (7%) in the low-inclusion group and a significant increase (14%) in the high-inclusion group. Kinley et al. [77] used freeze-dried *A. taxiformis* at inclusion rates of 0.05, 0.10, and 0.20% on an OM basis. While no significant effect on CH_4_ production was found in steers receiving 0.05% of alga, the authors reported a 40% and 98% reduction in methane production in the groups receiving 0.10% and 0.20%, respectively. No negative effect on DMI or FCE was observed, while the average daily weight gain (ADWG) increase by 26% and 22% was observed for 0.10% and 0.20% inclusion groups, respectively.

Li et al. [78] used air-dried and ground *A. taxiformis* and reported a CH_4_ production decrease of up to 80% with an inclusion rate of 3% on an OM basis. However, the authors reported refusals of *A. taxiformis* in the groups with inclusion rates of 2% and 3%, respectively. The actual intake of added material was calculated over an 11-day period with the results of 1.0–1.5% and 1.2–3.0% for the 2% and 3% groups, respectively (Table 2). No negative effect on DMI and LW was observed.

The data from different studies, although inconsistent, indicate that the inclusion of *Asparagopsis* spp. in ruminant diets can negatively affect DMI, which can consequently reduce animal productivity [73,74,78,79]. Several studies reported increased refusals or selection against *A. taxiformis* when it was used, indicating a low palatability of alga, possibly caused by a high concentration of minerals, as proposed by Roque et al. [73]. In addition, damage to rumen mucosa was reported in some studies [78,79], although it was not clear if the damage was caused by bromoform particularly. Glasson et al. [80] present a toxicological assessment of bromoform, in which large bolus doses of bromoform were used in rats, and argued that concentrations of CHBr_3_ in *Asparagopsis* spp. are negligible in terms of a possible negative impact on ruminant health. As for bromoform residues in animal tissues, the data from various studies support the observations of Glasson et al. [80]. It has been shown that bromoform does not accumulate in meat or fat [77,78,79], and only trace amounts can be found in milk [79]. Interestingly, Roque et al. [73] and Stefenoni et al. [74] reported trace amounts of bromoform in control groups also, and no significant difference was observed between the control and treatment groups. Stefenoni et al. [74] reported that the iodine amount was 7.8 times higher in the treatment than in the control group, which could pose risks to public health. The European Food Safety Authority (EFSA) recommends maximum levels of iodine of 2 mg I/kg feed for dairy ruminants and 10 mg I/kg feed for beef cattle [81], so strict precautions should be taken when formulating rations with red algae, especially for dairy cattle, as children can be particularly sensitive to elevated levels of iodine because of higher milk consumption [82,83]. In addition to high mineral and trace element content, red algae also accumulate heavy metals, as indicated by Selmi et al. [84], and, although in their study the reported levels of these elements were in the tolerable range, they could differ between geographical locations.

Vucko et al. [85] examined the effects of different processing techniques of *A. taxiformis* and concluded freezing and freeze-drying to be the most effective in preserving bromoform concentrations. However, a more recent study by Stefanoni et al. [74] showed that CHBr_3_ concentration in freeze-dried *A. taxiformis* decreased by 75% and 84% when stored in dark and light conditions, respectively. The authors reported that the temperature of storage was not a significant factor. The data from this experiment prove a need for better processing techniques of red seaweed to achieve better stability of bromoform, as large-scale processing and distribution will also most probably be lengthy processes, at least in some regions. Furthermore, bromoform concentrations in red seaweed are affected by factors such as habitat, species, strain used, light intensity, stage of lifecycle, water temperature, nutrient availability, etc. [69,77]. This warrants more research to establish the optimal growing, harvesting, and subsequent processing methods to reduce the variability in the bromoform concentration in *Asparagopsis* spp.

Even though CHBr_3_ is a compound that can deplete the ozone layer, it is classified as a very short-lived substance (VSLS) with an average lifetime of 24 days [86] and therefore has low potential for ozone depletion. However, large-scale production and processing of red seaweed might increase bromoform emissions and it should be considered when creating emission models and extrapolating the current data. Upstream CO_2_ emissions resulting from such production systems should also be evaluated.

In conclusion, the red seaweed genus *Asparagopsis* is a very promising strategy to reduce ruminant CH_4_ emissions, but currently more research is needed to determine the sustainability and viability of the implementation of this strategy on a large scale.

#### 2.3.2. Tannins

Tannins are water-soluble compounds of high molecular weight found in various parts of plants. These are plant secondary metabolites, the purpose of which is to protect the plant from insects, bacteria, or herbivores [87,88]. Tannins (TAs) are generally divided into two groups—lower-molecular-weight hydrolyzable tannins (HTs) and larger condensed tannins (CTs)—with some authors distinguishing a third group, which is found mostly in algae—phlorotannins [89,90]. Although both main groups of tannins can be toxic, hydrolyzable tannins, due to their lower molecular weight they have poorer adsorption to proteins [91]. Consequently, more HTs undergo microbial hydrolysis, and the resulting metabolites can be toxic [92]. Condensed tannins have a stronger effect on the rumen metabolism and microbiota activity and generally carry lower toxicity potential than hydrolysable tannins, and thus are more attractive as possible methanogenesis mitigators [93,94]. The exact mechanisms of the antimethanogenic properties of CTs have not yet been clearly defined, but their effect on methanogenesis could be explained by both direct and indirect modes of action [42,94,95,96]. The direct effect of tannins is most likely explained by the ability of tannins to bind to the cell envelope of archaea, thus impairing the establishment of the methanogen–protozoa complex, decreasing interspecies hydrogen transfer, and inhibiting methanogen growth [94,97], while the indirect effect is largely based on toxicity to bacteria and protozoa, which produce the H_2_ needed for methanogenesis [20,58,98]. Some of the indirect effects could also be explained by their binding activity to proteins and polysaccharides, thus reducing nutrient availability.

The molecular weight, structure, activity, and concentration of tannins vary greatly depending on the species, age, and the part of the plant used, so the results of the tests carried out with tannins in the literature are variable. This is understandable considering the broad group of compounds that tannins are. Yang et al. [99] conducted a study with beef cattle using 6.5 g, 13 g, and 26 g TA/kg DM and found an 11.1, 14.7, and 33.6% decrease in CH_4_ production (L/kg of DM consumed), respectively. The authors also observed a decrease in relative protozoa and methanogen abundance and consequently reduced OM digestibility at 26 g TA/kg DM. Crude protein digestibility was reduced by 5, 8.6, and 15.7% for 6.5, 13, and 26 g TA/kg DM, respectively. Consequently, 13 and 26 g TA/kg DM reduced ammonia nitrogen (N-NH_3_) in the rumen. Orzuna-Orzuna et al. [100] included 32 trials with beef cattle in their meta-analysis. In different trials, CTs and HTs were used separately and together. It has been observed that an average addition of 14.6 g tannins/kg DM to diets reduced methane production by an average of 9.89%. A decrease in DM digestibility was reported when the concentration of TAs exceeded 12 g/kg DM, but no effect on average daily weight gain (g/d) was observed. N-NH_3_ concentrations in rumen were decreased and a nitrogen shift from urine to feces was observed. This is a positive result since nitrogen excreted in urine is less stable and results in higher N_2_O emissions than nitrogen excreted in feces [91,100]. Valencia Salazar et al. [101] achieved a reduction of 50.9% in L CH_4_/day with the incorporation of *Samanea saman* pod meal (30% of DM) into a crossbred heifer ration without affecting DMI or apparent DM digestibility. The authors did not observe any reduction in protozoal count. However, a part of the significant reduction in CH_4_ emissions was attributed to the presence of saponin in *S. saman* pods. In their study with sheep, El-Zaiat et al. [102] used *Acacia saligna* and *Leucaena leucocephala* and reported 12.3 and 10.5% reductions in CH_4_ production (L/d), respectively. Of the two plant species used, *Acacia saligna* increased fecal N, retention N, and reduced urinary N without negatively affecting DMI or apparent digestibility.

Tannins in the rations containing the amount of CP above the requirement could be beneficial and increase animal productivity while also decreasing ammonia excretion, but in mid-quality or poor diets, their inclusion would probably be harmful [103]. Compounds of this group together with positive effects might also cause negative effects, i.e., a decrease in palatability and/or digestibility, toxicity, etc. Generally, tropical plant species contain higher amounts of TAs than temperate climate plants and, consequently, tropical plants exert stronger effects on ruminant metabolism. Tropical plant species, even if found to be effective, will not likely be used in temperate zones extensively, particularly because of the challenges of cultivating such plants in colder climates. Extracts of tannins from specific plants could be commercialized, but in some instances (e.g., tannins from bark or tree leaves) the collection of material can be costly and labor-intensive. Tanniferous plants, which can be easily harvested for further processing or feeding feedlot animals, e.g., birdsfoot trefoil (*Lotus corniculatus* L.), seem most likely to be implemented in large operations. Tanniferous plant use is particularly attractive for pastoral production systems, as a limited number of strategies are adoptable in such systems. Furthermore, because of the anthelminthic properties of tannins, the use of specific tanniferous plants for grazing could additionally benefit small ruminant operations by improving animal productivity and would possibly decelerate the development of anthelmintic resistance. More research is needed to identify the most promising tanniferous plants and to evaluate their possibility to be used for CH_4_ mitigation in different regions of the world. In conclusion, the use of tannins or tannin-containing plants is a compelling and sustainable strategy, but due to their variable efficacy they are most likely to be used in conjunction with other methane emission mitigation options.

#### 2.3.3. Saponins

Saponins are another group of plant secondary metabolites consisting of various glycosides [104]. It is thought that the main antimethanogenic effect of saponins is indirect, i.e., by emulsifying the cell walls of rumen protozoa, saponins disrupt their permeability and thus cause cell death. As protozoan populations decrease, the amount of H_2_ required for methanogenesis decreases accordingly. Kozlowska et al. [105] found a decrease in methanogen populations along with a decrease in protozoan populations during in vitro trials, so the possibility of a direct toxic effect of saponins on archaea cannot be ruled out, although the reduction in archaeal populations could also be attributed to the decrease in the protozoal count because of interrupted interspecies H_2_ transfer.

Jayanegara et al. [106] carried out a meta-analysis comprising 23 studies where different saponin sources were used. The saponin-rich plants used were yucca (*Yucca schidigera*), soap bark tree (*Quillaja saponaria*), and tea (*Camellia sinensis*). The authors reported that the addition of increasing levels of a saponin-rich source decreased methane emission per unit of substrate incubated as well as per unit of total gas produced. The proportion of propionate increased with the increasing levels of saponins and protozoal count decreased.

Goel and Makkar [107] analyzed 12 in vivo trials with saponins, 7 of which reported a decrease in CH_4_ production from 6% up to 27%. Zhang et al. [108] achieved 28, 35.8, and 47.9% reductions in methane production using tea seed saponin in sheep ration at inclusion rates of 5, 10, and 20 g/kg DM, respectively. Furthermore, apparent CP digestibility was increased with an inclusion rate of 20 g/kg DM. Albores-Moreno et al. [109] fed saponin-rich ground pods of guanacaste (*Enterolobium cyclocarpum*) to hair sheep. The amounts of ground pods fed were 0.15, 0.3, and 0.45 kg with saponin contents of 4.35, 8.70, and 13.05 g, respectively. The results showed a tendency for increased DMI with increasing doses of saponin. Methane production was reduced by 21.1, 36.6, and 26.1% with saponin inclusion rates of 4.35, 8.7, and 13.05 g/animal/day, respectively. However, the total methane reduction effect could not be fully attributed to saponins only, as nutrient composition and other plant secondary metabolites of ground pods could have affected methanogenesis. Part of the antimethanogenic effect could also be explained as a result of increased DMI as the inclusion rate of ground pods increased. Increased DMI may have consequently increased the digesta passage rate, thus reducing nutrient availability for rumen microbiota. Mao et al. [110] used tea seed saponin at 3 g/animal/day and reduced methane production in growing lambs by 27.7%. Interestingly, the authors observed decreased protozoa populations with no effect on methanogen populations. Liu et al. [111] fed tea saponin at 2 g/sheep/d and reported increased apparent OM digestibility together with an increased proportion of propionate. Consequently, absolute methane production was not affected, but the effect was apparent when scaled to metabolic body weight (8.8% decrease).

Kozlowska et al. [105] reported that saponins from different parts of alfalfa varieties (*Medicago sativa* L.) could also be used in methane emission mitigation efforts. The efficacy of alfalfa was also proven in vivo by Kumar et al. [112] in a study with sheep. The authors observed 5.70, 6.46, and 22.62% reduced daily methane production when alfalfa hay was included to provide saponins at 100, 200, and 400 mg/kg BW. A reduction in the protozoal count was also reported. Malik and Singhal [113] reported 21% lower methane production in buffaloes which were fed with alfalfa hay replacing 30% of the diet together with a reduction in the protozoal population by 20%. It could also be hypothesized that part of the methane-mitigating effect in the aforementioned two studies was achieved because alfalfa is a plant of higher quality and nutritional value, but the reduction in protozoal count might be a strong indicator of disrupted interspecies H_2_ transfer between protozoa and methanogenic archaea. However, in a study by Kumar et al. [112], the lowest protozoal count was observed on day 3 of the treatment and slowly increased following consequent counting on days 7, 14, and 38 in treatment groups of 200 and 400 mg/kg BW inclusion. One of the possible explanations for the alteration in the protozoal count during the trial could be that saponins undergo deglycosylation faster as ruminal bacteria might adapt to higher amounts of saponin present [114,115]. As for methane emission, measurements were performed after a 4-week feeding trial so no data are available to determine if methane production was correlated with shifts in protozoal count during the trial.

Ramírez-Restrepo et al. [116] fed tea seed saponins (20 g/animal/d later increased to 30 g/animal/d) to cattle but did not observe any statistically significant reduction in methane production. Conversely, the authors reported an increase in the protozoal count, which was rather unexpected. In addition, no antimethanogenic effect was observed when saponins were included in the diet, but methane reduction was reported after the saponin feeding period. Guyader et al. [117] reported a decrease in methane production in vitro, but the same effect was not achieved in vivo. Methane yield increased as a result of decreased DMI due to saponins (inclusion rate of 0.52% DM). The protozoal count was not significantly affected. In their previous study with dairy cattle, Guyader et al. [118] also reported no effect on CH_4_ emission at a tea saponin inclusion rate of 0.5% DM.

The results obtained with saponins, same as with tannins, vary greatly and can also be attributed to differences in the plant species used, variations in the rate of inclusion, different animal type, and diet composition. Nonetheless, as reported in some studies, saponins do have a potential for enteric methane emission mitigation and various species of saponin-rich plants can be used widely in different geographical locations. Moreover, the addition of pure saponin to rations could also be facilitated, as, for example, tea seed saponins are considered a byproduct of tea seed oil extraction [110]. However, due to the possible short-term effects of saponins [114,119], they might be unlikely to be used as a sole mitigation strategy, but more research is needed to elucidate the long-term effectivity of saponins. Other sources of saponins, such as alfalfa, are already widely used because of their nutritional value and apparently can reduce both methane production and intensity. Tannin- and saponin-containing plant species, e.g., birdsfoot trefoil and alfalfa, could be used in tandem to examine the potential of their additive effect on methanogenesis both in grazing-based production systems and as dried or ensiled forage mix.

#### 2.3.4. Essential Oils

Essential oils (EOs) are the third group of plant secondary metabolites reviewed in this article. The main functions of EOs are to protect plants from abiotic stress, infections, damage, and pests [120]. These metabolites in plants often determine their smell and color, so they are used in cosmetics, perfumery, and pharmaceuticals [42]. The main active compounds of essential oils usually belong to two main groups—terpenoids and phenylpropanoids [121]—but the composition of EO also includes, although in lower concentrations, substances such as alcohols, acids, acyclic esters, aldehydes, etc. [122]. Because of their antimicrobial properties, essential oils affect ruminal microbial populations and have the potential to reduce methane emissions [123].

The antimicrobial effect of essential oils is associated with membrane damage, changes in electron flow and ion gradient, protein translocation, and phosphorylation [124,125]. Although the exact mode of action on methanogenesis has not yet been elucidated, it is thought that essential oils, due to their broad-spectrum antimicrobial activity, can influence methanogenesis both directly, via toxic effects on methanogenic archaea, and indirectly, via toxic effects on cellulolytic bacteria and protozoa, thereby reducing the amount of H_2_ available for methanogenesis [123]. Chao et al. [126] found that although there are essential oils with a wider spectrum of activity, these compound mixtures are more effective on gram-positive bacteria than other microorganisms. Extensive research proved some EOs to be effective in vitro but lack of consistency or effect was reported in vivo.

As with other plant secondary metabolites, the vast diversity of EOs leads to different results. Soltan et al. [127] in their study with sheep used a microencapsulated blend of essential oils consisting of cinnamaldehyde, eugenol, carvacrol, and capsicum oleoresin. The authors reported 27.7 and 35.9% reduced CH_4_ production (L/kg digestible organic matter), compared to control, at inclusion rates of 200 mg and 400 mg/kg DM, respectively. The enhanced effect on methanogenesis with an EO dose of 400 mg/kg DM could be partially attributed to a significantly decreased protozoa count, which occurred 1 week after the beginning of supplementation. No significant differences were observed on DMI or total tract nutrient digestibility. Both treatments also reduced the acetate/propionate ratio.

Santos Torres et al. [128] analyzed 11 studies using various essential oils in trials with sheep and found no statistically significant effects on methanogenesis or rumen fermentation parameters. Wang et al. [129] used eucalyptus and anise oils for sheep at 0.5 g/animal/day and also reported no effect on methanogenesis, although a tendency (*p* = 0.08) for anise oil to reduce methane emission was noted.

In their study with buffaloes, Yatoo et al. [130] used a blend of ajwain (*Trachyspermum ammi*), garlic (*Allium sativum*), and cinnamon (*Cinnamomum verum*) leaf oils at inclusion rates of 0.15 and 0.3 mL/kg DMI. The authors reported no effect on absolute methane emissions per day, but CH_4_ production expressed as L/kg DMI was reduced by 14.1 and 14.2% in both groups, respectively. No effect on nutrient utilization was observed, but the authors reported an increased DMI in both treatment groups with a tendency for increased body weight gain. It could be speculated that the increased DMI in the treatment groups resulted from an increased palatability/aromaticity of the feed when the EOs were added, as the oils used were not encapsulated. A tendency for an increased body weight gain could confirm this, as no effect on the digestibility of nutrients was observed. Conflicting results were published by Alemu et al. [131], who reported an 11% increased daily CH_4_ production g/d when supplementing steers with an EO and pepper extract mix Activo^®^ Premium. No effect on the average DMI, ADG, or gain-to-feed (G:F) ratio was observed. In their meta-analysis of 10 studies, Torres et al. [132] reported no effect on methane production in beef cattle fed high-concentrate diets. In addition, the authors observed an increased prevalence of hepatic abscesses by 84.9%, which highlights the risks of health issues that may arise in different production systems. Nigel Tomkins et al. [133] found no reduction in methane production in trials with a commercial essential oil blend CRINA^®^ (mixture of thymol, eugenol, vanillin, limonene, and guaiacol) supplemented to beef cattle.

Chaouki Benchaar [134] used oregano (*Origanum vulgare*) essential oil with carvacrol as the main active component and reported no effect on methane production in dairy cattle. Belanche et al. [135] conducted a meta-analysis of trials with the essential oil blend Agolin Ruminant^®^ and found an average 8.8% reduction in methane production (Table 3).

The effects of different essential oils and/or their blends on methanogenesis are highly variable. The results are dependent not only on the properties of EOs but also on the animal type, composition of rations, and duration of the trial. The use of EOs might not be suitable in some production systems such as high-concentrate beef cattle operations, because of potential risks to animal health. It is challenging to indicate which EOs are the most effective, as the types and doses of EOs differ in the studies. Such discrepancies warrant more in vivo research to pinpoint the most effective essential oils, which then could be tested more intensively to determine appropriate concentrations. The possible additive/synergistic effect of certain different essential oils encourages scientists to study essential oils as blends rather than singular substances [123,136]. In conclusion, even though there is a wide variation in the results, the use of essential oils shows potential in mitigating enteric methane emissions. Furthermore, the natural origin of EOs promotes research on the potential of their use in animal husbandry because of higher consumer acceptance and the ongoing general shift towards ecological and sustainable farming practices.

#### 2.3.5. Cyanogenic Glycosides

Cyanogenic glycosides (CGs) are a group of natural compounds found in certain plants and act as a defense mechanism against herbivores [137]. Chemically, CGs are defined as O-β-glycosides of cyanohydrins that form as a result of cyanohydrin glycosylation. CGs are amino acid derivatives and, in nature, their only precursors are l-valine, l-isoleucine, l-leucine, l-phenylalanine, l-tyrosine, and cyclopentenylglycane [138]. Cyanogenic glycosides are present in more than 2650 plant species, but can be found in higher concentrations in such plants as sorghum (*Sorghum* spp.), certain clovers (*Trifolium* spp.), arrowgrasses (*Triglochin* spp.), and cassava (*Manihot esculenta*) [139]. These bioactive compounds, depending on plant species, can be stored in the leaves, stems, seeds, or roots of plants. Generally, CG concentrations are higher in younger plants but environmental factors, such as frosts or droughts, can prompt more intense synthesis of these substances in more mature plants. Cyanogenic glycosides are most commonly stored in the vacuoles of plant cells and are inactive on their own. The other component needed to activate the defense mechanism is the enzyme β-glycosidase, which is stored in other cell compartments to prevent autotoxicity [140]. During plant maceration, β-glycosidase quickly comes into contact with the CG and splits it into a sugar molecule and a cyanohydrin, which is then further hydrolyzed, resulting in the formation of hydrogen cyanide (HCN) [141]. HCN binds to cytochrome oxidase, thus blocking mitochondrial respiration [142]. HCN is considered an extremely toxic substance and poses even more risk to ruminants because the abundance of microflora in the rumen and a favorable pH permit faster hydrolyzation of CGs in a ruminant than in a monogastric animal [143].

The toxicity of cyanide to methanogens has been known for a least 4 decades and was a problem encountered in anaerobic wastewater treatment systems [144,145,146,147]. The inhibitory action on archaea is explained by cyanide ions’ ability to tightly bind to metal-containing proteins and enzymes, which are present in anaerobic microflora and abundant in archaea. Thereafter, the mode of action of cyanide is at least in part identical to that of halogenated hydrocarbons, which are discussed in another section of this article.

The research focused on cyanide as a candidate for inhibiting enteric methane formation consists mostly of trials performed in vitro. Zavaleta et al. [148] performed an in vitro trial with CG linamarin (abundant in cassava) inclusion rates of 6, 13, 20, and 26 mg/L and reported a decrease in methane production by 9.7, 9.2, 18.1, and 29.4%, respectively. Phanthavong et al. [149] used ground leaves of sweet and bitter cassava varieties and reported a 23.6% lower total methane volume using the bitter cassava variety compared to the sweet variety. Authors attributed the effectiveness of the bitter variety mainly to the higher CG content present in these plants. Phuong et al. [150] carried out an in vivo trial with goats fed on both bitter and sweet versus sweet varieties of cassava. The researchers reported a 16% lower methane/carbon dioxide ratio when the goats were fed both varieties compared to the sweet variety alone. Feeding both varieties also increased DMI and N retention by 25 and 23%, respectively. No indications of cyanide toxicity were observed, although thiocyanate levels in urine increased by 100% in the mixed variety group compared to the sweet variety group.

Cyanogenic glycosides can be effective in reducing enteric methane emissions, but this topic warrants more intense research to determine which CGs are the most suitable for such use, as well as their dose and mode of administration (plant material or pure encapsulated compounds, slow-release formulations, etc.) because relatively small amounts of cyanide can be toxic [143,151]. Most importantly, for this strategy to be implemented on larger production systems, long-term effects on ruminant health parameters should be investigated more thoroughly.

### 2.4. Alternative Electron Acceptors

#### Nitrates and Sulfates

Some of the rumen microorganisms are able to reduce nitrate (NO_3_^−^) and sulfate (SO_4_^2−^), with the formation of ammonium ions (NH_4_^+^), nitrogen (N_2_), and hydrogen sulfide (H_2_S) as end products.

**Figure 1:**
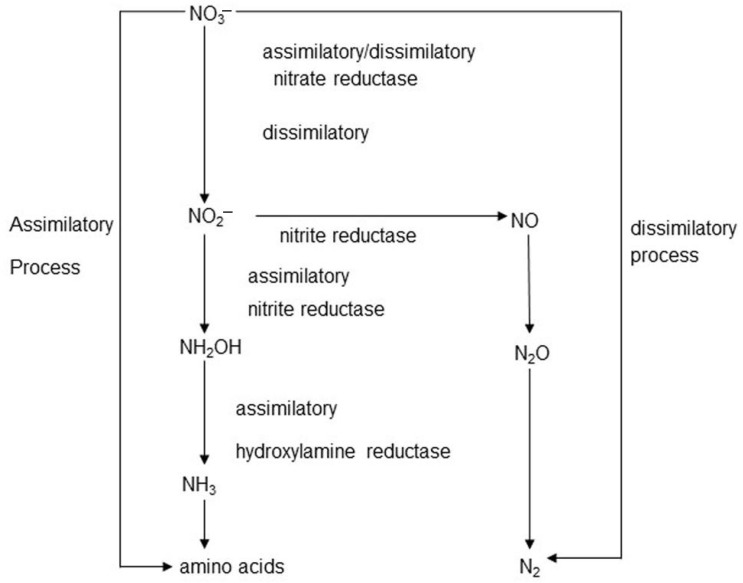
The assimilatory and dissimilatory routes of nitrate/nitrite metabolism. Adopted with permission from Yang et al. [157].

Nitrate reduction in the rumen can take place via assimilatory and dissimilatory pathways (Figure 1). The final product of both of these processes is NH_4_^+^, but assimilative nitrate reduction uses energy to produce ammonium, while dissimilatory reduction is an energy-generating process. Ammonium formed during reduction can be used as a nitrogen source for microbial protein synthesis [152,153]. Ammonium later converts to ammonia (NH_3_), but the balance between these two compounds is highly affected by pH, with ammonia concentrations increasing as pH increases [154]. Denitrification is also a dissimilatory nitrate reduction pathway, the intermediate product of which is nitrous oxide (N_2_O) and the final product is nitrogen (N_2_). Although nitrous oxide is a very potent GHG, there are relatively few genes encoding denitrification in rumen metagenomics samples, so this process is not considered to be a very significant pathway for nitrate metabolism in the rumen, although it cannot be ruled out [155,156].

**Figure 2:**
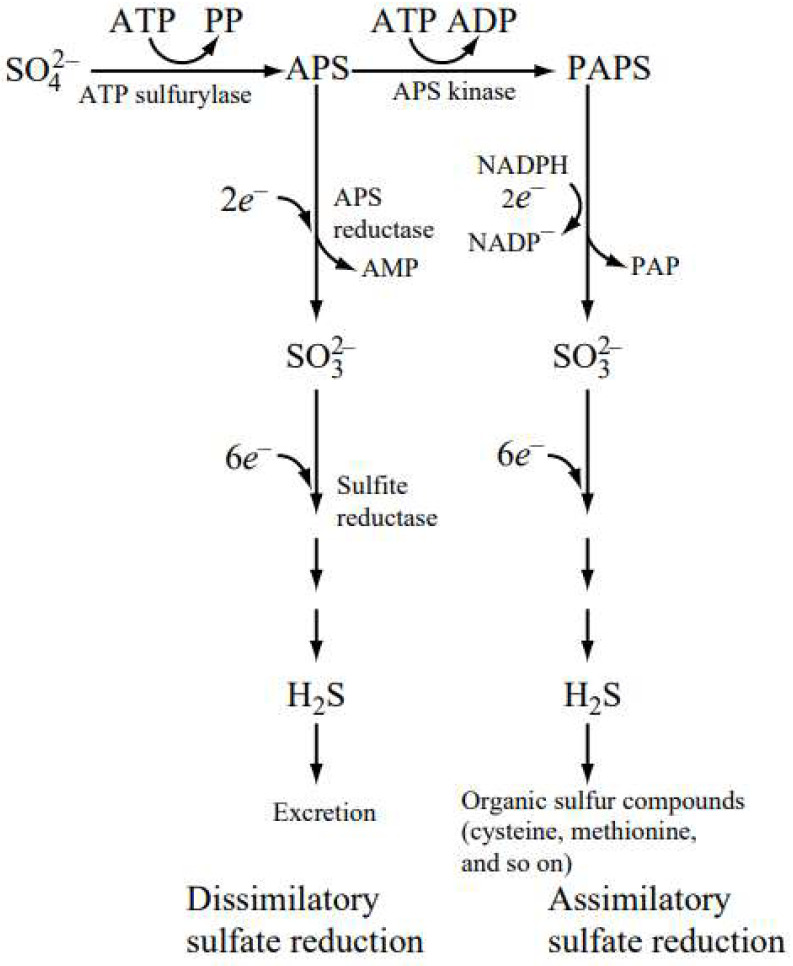
Dissimilatory and assimilatory route of sulfur reduction in rumen. Adopted with permission from Goldhaber [159].

Bacterial sulfate reduction is also divided into assimilatory and dissimilatory (Figure 2). During assimilatory reduction, bacteria reduce sulfate to H_2_S and incorporate sulfur (S) in the synthesis of S-containing amino acids and coenzymes [158]. During dissimilatory reduction, hydrogen sulfide is the final product of bacterial metabolism. At pH 6.5, 87% of H_2_S is dissociated into sulfidic anions (HS^−^) and remains in the rumen liquid phase; the other part is released as gas, which is expelled from the rumen via eructation.

Microorganisms can use H_2_ as an electron donor for the reduction of both NO_3_^−^ and SO_4_^2−^. Nitrate and sulfate reduction are thermodynamically more favorable reactions than methanogenesis, so these compounds can act as hydrogen sinks, thus lowering the amount of H_2_ available for methanogenesis [160].

The reduction of nitrite to ammonium in the rumen is a slower process than the reduction of nitrate to nitrite. Nitrite is readily absorbed into the bloodstream from the rumen, so the accumulation of nitrite may put the animal at risk of methemoglobinemia [161]. The potential toxicity can be reduced by gradually increasing the amount of nitrate in animal diets so that the colonies of nitrate- and nitrite-reducing bacteria can adapt to the increasing amount [157]. Another method is to use sulfates together with nitrates in the rations. The final product of sulfate reduction, hydrogen sulfide, can act as a potential electron donor in the process of reducing nitrite to ammonium, thus shortening the residence time of the toxic compound in the rumen [162]. H_2_S is known to be toxic [163], so its oxidation in the rumen would also be beneficial.

Almeida et al. [61] analyzed 25 studies in which the dose of nitrate used ranged from 17.2 to 22.2 g/kg DM. A decrease in methane yield was observed ranging from 10 to 22.1%. DMI and fiber digestibility were not affected. Van Zijderveld et al. [161] reported a 32% reduction in methane production in sheep after feeding nitrate at a 2.6% DM basis. They also observed a 16% decrease in CH_4_ production after the introduction of sulfate at 2.6% on a DM basis. The scientists also noticed that when nitrate and sulfate were used together, their anti-methanogenic effect was cumulative—methane production decreased by 47%. No effect on feed intake or average weight gain was observed. Feng et al. [164] included 24 studies and reported an average decrease in CH_4_ production of 20.4 and 10.1% for dairy and beef cattle, respectively, with an average NO_3_^−^ inclusion rate of 16.7 g/kg DM (Table 4). It should be noted that the difference in the reduction in methane production is also attributed not only to the cattle type but also to the type of administration of NO_3_^−^ (beef cattle mostly received slow-release nitrate).

Overall, the use of nitrate and sulfate seems like an effective methane emission mitigation strategy, but care should be taken when administering these substances as the metabolism of both compounds results in toxic intermediate or end products (NO_2_^−^ and H_2_S). One of the possible ways to reduce the risk of toxicity could be microencapsulating nitrate, which could help to provide more stable delivery to the rumen. For both nitrate and sulfate, the administration of nitrate-, nitrite-, and sulfate-reducing bacteria as probiotics would help to reduce the risk of toxicity for the animal. Different nitrate and sulfate salts and/or their combinations should be tested in vivo in search of the most effective mix (e.g., sodium and potassium nitrate is more effective than calcium nitrate). In addition to methane reduction potential, nitrate is a non-protein nitrogen (NPN) source and could benefit animal production systems where dietary CP content is insufficient. However, in the production systems where the dietary CP in rations is high, feeding nitrate can increase nitrogen concentration in manure/urine, which would result in increased N_2_O emissions. Furthermore, in animal diets where distillers grain is used (high S content), additional sulfate inclusion is likely to increase the risk of disorders such as polioencephalomalacia.

### 2.5. Probiotics

The use of probiotics in animal feed is already quite popular, although for other reasons. Probiotics, also known as direct-fed microbials (DFMs), are microbiological feed additives based on selected bacterial or yeast cultures [165]. Probiotics can enhance ruminal fermentation, improve feed digestibility, and regulate the growth of pathogens [166,167,168,169,170]. DFMs could also help to reduce ruminant methane emissions by competing for H_2_, shifting fermentation pathways, and enhancing other microorganisms which would reduce the amount of hydrogen available for methanogens or simply protect the rumen microbiota and/or the animal itself from undesired effects of feeding different additives, such as NO_3_^−^.

Yeast, such as *Saccharomyces cerevisiae*, can improve the general health of animals, feed consumption, and fiber digestibility, which results in an increase in milk production or body weight gain. Although the benefits of yeast for ruminants have been confirmed by a number of scientific studies, yeast is probably not the right strategy when it comes to methane emissions. Harper et al. [171] fed lactating cows with probiotics based on *Saccharomyces cerevisiae* and reported no significant effect on CH_4_ production, intensity, or yield. However, milk yield was increased by 2 kg/d in the treatment group, with no effect on DMI or feed digestibility. Darabighane et al. [172] performed an analysis of 46 studies published from 1990 until 2016 and reported no significant effect on CH_4_ production. Although no direct effect on methane emission was observed, improved animal production might result in lower methane intensity and, consequently, lower the total methane footprint of animals in certain production systems, e.g., faster-growing meat breeds could reach target weight faster. However, since no direct effect on methanogenesis was observed, this option will not be discussed further. Different species, strains, and doses could be used to screen for possible candidates for enteric methane mitigation.

Lactate-producing bacteria (e.g., *Lactobacillus*, *Enterococcus* spp.) are often used in ruminant feeding. Due to constantly elevated levels, the microflora of the rumen adapts to the presence of lactic acid, thus also reducing the risk of acidosis [22]. Doyle et al. [173] proposed three distinct ways to explain how lactate-producing bacteria can influence methanogenesis:

1. Bacteria or their metabolites shift rumen fermentation and consequently reduce methanogenesis;
2. Bacteria or their metabolites directly inhibit rumen methanogens;
3. Bacteria or their metabolites inhibit H2-producing microorganisms.

Jeyanathan et al. [174] used *Lactobacillus pentosus* D31, *Lactobacillus bulgaricus,* and *Propionibacterium freudenreichii* 53-W in their trial with sheep. The authors reported no effect of *Lactobacillus bulgaricus* on CH_4_ emission, while *Lactobacillus pentosus* D31 reportedly decreased methane production by 13%. Interestingly, *Propionibacterium freudenreichii* 53-W increased CH_4_ production by 16%. In their later trial with dairy cattle, Jeyanathan et al. [175] also reported a 27% increased CH_4_ intensity when *Propionibacterium freudenreichii* 53-W was added to the feed. Lactate-utilizing bacteria (LUB) such as *Propionibacterium* spp. and *Megasphaera* spp. can increase the production of propionate in the rumen, thereby increasing the amount of energy available to the ruminant and reducing the risk of acidosis by reducing the concentration of lactate in the rumen. Propionate production is also an alternative electron sink, diverting hydrogen flow from methanogenesis [176], so lactate-producing and lactate-utilizing bacteria are often used in tandem to promote propionogenesis [174]. However, in the trials conducted by Jeyanathan et al. [174,175], no changes in the VFA profile were detected, suggesting other effects of DFM on CH_4_ mitigation. The increase in methane production could be due to the direct effect of the strain introduced into the diet on methanogens or an indirect effect through metabolites affecting the ruminal ecosystem [173].

Naturally found in rumen cultures, acetogenic bacteria can use hydrogen to produce acetate during a process called reductive acetogenesis, which in theory can compete with methanogenesis. However, the reduction of CO_2_ by hydrogen to CH_4_ is a thermodynamically more favorable reaction than the reduction of CO_2_ to acetate [8,177]. To successfully compete with methanogens, acetogenic bacteria require a higher H_2_ partial pressure in the rumen, so in this case, natural cultures of acetogens in the rumen act more as a protective mechanism, which is activated more intensively when methanogenesis stops and the hydrogen concentration increases rapidly. Such bacteria can be useful for reducing increased hydrogen concentration when effective methanogenic population control methods are applied. Because of the higher affinity of methanogenic archaea to H_2_ and a consequent need for higher hydrogen concentrations in order to be effective, reductive acetogenesis so far seems like an unlikely method to effectively reduce methane emissions.

Probiotics have the potential to aid in methane emission mitigation efforts when used in conjunction with other strategies. One such possibility is using NO_2_-reducing bacteria. As discussed in the previous section, nitrate has the potential to reduce methane emissions by competing with methanogens for H_2_, but the toxic intermediate—nitrite—poses health risks for animals. An additional inclusion of nitrite-reducing bacteria in the rations of steers supplemented with nitrate was proven to be effective—Latham et al. [178] reported that *Paenibacillus* 79R4 aided nitrite metabolism and prevented methemoglobin accumulation. Other classes of microorganisms, such as sulfate-reducing or lactate-utilizing bacteria, can help minimize the negative effects associated with certain mitigation strategies.

Other microorganisms, such as *Bacillus licheniformis*, can positively affect ruminal fermentation and reduce CH_4_ emissions via pathways that have not been clearly elucidated yet. Deng et al. [179] fed probiotics based on *Bacillus licheniformis* to sheep and reported an average 12% reduction in methane production when scaled to digestible DM for low- and medium-inclusion rates of the additive—2.5 × 10^8^ and 2.5 × 10^9^ colony-forming units (CFU) of *B. licheniformis* per head per day, respectively. A slightly smaller, 9.3%, reduction was observed in the high-inclusion group (2.5 × 10^10^ CFU) (Table 5). The addition of *Bacillus licheniformis* also improved apparent nutrient digestibility, and improved the N utilization efficiency and energy metabolizability. The authors postulated that the possible mechanisms of action of *Bacillus licheniformis* might include secretion of extracellular enzymes, immunomodulation, antimicrobial production, and competitive exclusion.

Due to the particularly wide variety of microorganisms and their different abilities to influence the fermentation process, the possibilities of using probiotics have a lot of potential not only in methane mitigation but in enhancing animal health and productivity in general. Probiotics, when used in tandem with other additives used for emission mitigation, demonstrate the ability to enhance their effects or reduce potential risks associated with before-mentioned additives. However, care should be taken to investigate the use of different species and strains of microbes, as some have been shown to increase methane emissions. Due to the wide possibilities of application, more research is needed to screen for such microorganisms and their interspecies connection. Overall, probiotics seem a very likely option to reduce CH_4_ emissions and can be used together with other mitigation strategies.

## 3. Conclusions

Currently, some of the most widely studied enteric methane mitigation strategies are the use of 3-NOP, nitrates, plant secondary metabolites (PSM), and macroalgae, out of which *Asparagopsis taxiformis* and *Asparagopsis armata* are the main seaweeds studied.

3-NOP was proven to be an effective emission mitigation strategy, but it is likely to be applied mostly in confined systems such as feedlots where it can be incorporated into the animal feed. This strategy seems to be difficult to adopt in grazing-based production systems for the reason mentioned before. It could be speculated that the creation of a slow-release capsule or bolus might be one of the possible solutions. Furthermore, in the European Union, 3-NOP is authorized only for use for dairy cattle, but it is highly likely that authorization will include other ruminants, such as sheep and goats, in the near future.

Although proven to be very effective, the use of seaweeds raises some issues. Firstly, two of the most effective seaweed species are found mostly in tropical waters, so their production in colder waters is questionable. Some companies are exploring possibilities to create inland seaweed production systems, but such systems are expected to be energy-consuming (maintaining a constant temperature and salinity of water, filtration, etc.). Large-scale production of seaweed for ruminant feeding will result in additional upstream emissions from different production stages and care should be taken to estimate if the carbon footprint of such industry will not offset the reduction coming from feeding the seaweed. It should be stressed that the main active component of red seaweed, bromoform, although short-lived (24 days), is a compound with ozone-depleting potential and improper processing of seaweed can increase bromoform flux into the atmosphere. Furthermore, it has been proven that bromoform content in seaweed degrades substantially over time, so other processing methods than freeze-drying should be explored.

The use of nitrates and sulfates, although effective, poses some risks to animal health. Nonetheless, current research suggests that these risks can be minimized or circumvented. No commercial products of these compounds formulated specifically for ruminants exist to the author’s knowledge. To help minimize potential risks, encapsulation or other techniques should be further explored to determine the safest and most effective methods of administration. The addition of specific direct-fed microbials has also been proven to alleviate the risks arising from the usage of such compounds.

Other feeding strategies, such as the use of plant secondary metabolites, have also been proven to be effective. Regarding pasture-based systems, plants containing higher amounts of different secondary metabolites seem to be the most attractive methods to mitigate emissions from such systems. Additionally, the use of PSM also has the potential to contribute to the control of ruminant gastrointestinal parasitism and slow down the formation of anthelminthic resistance. Although the efficacy of such natural compounds on enteric methane formation is variable, their additive/synergistic effect warrants more research to find the most suitable plant or active compound combinations and doses. Furthermore, because of higher public acceptance, their natural origin, and the occurring shift towards ecological farming practices, certain plant species and their secondary metabolites will most likely become a part of a multilevel enteric emission mitigation plan.

Due to various geographical and agro-ecological factors and adaptability issues, mass adoption of only one or two mitigation strategies is unlikely and a number of different strategies will probably be adopted in ruminant production systems across the world. In addition, the possibility of a combination of different mitigation strategies and their interactions should be assessed further.

## Figures and Tables

**Table 1:** Summary of the data on the effect of different synthetic compounds used for enteric methane mitigation.

Mitigation Strategy	Animal Type	Dose	Effect on Emissions	Reference
3-NOP	Beef cattle	100–200 mg/kg DM	−12–84% CH_4_P	[43]
3-NOP	Beef cattle	125 mg/kg DM	−70% CH_4_P	[44]
3-NOP	Beef and dairy cattle	N/A	−29–47% CH_4_P	[23]
3-NOP	Dairy cattle	60 mg/kg DM 80 mg/kg DM	−28.2–38% CH_4_P −31.4–45.1% CH_4_P	[45]
3-NOP	Dairy cattle	60 mg/kg DM	−26% CH_4_P	[46]
3-NOP	Sheep	100 mg/animal/day	−25% CH_4_P/kg DMI	[47]
Monensin	Beef cattle Dairy cattle	32 mg/kg DMI 21 mg/kg DMI	−19 g/d CH_4_P −6 g/d CH_4_P	[59]
Monensin	Dairy cattle	24 mg/kg DMI	No effect	[60]
Monensin	N/A	N/A	−4% CH_4_Y	[61]
Monensin	Beef cattle	150 mg/animal/day	No effect	[58]
Monensin	Dairy cattle	471 mg/animal/day	No effect	[62]
Monensin	Sheep	40 mg/kg DM	−12.7% CH_4_P	[63]
HHC (CF)	Dairy cattle	1.5 mL/animal/day	−38% CH_4_P	[66]
HHC (BCM)	Beef cattle	0.3 g/100 kg BW	−50–60% CH_4_P	[67]
HHC (BCM)	Dairy goats	0.3 g/100 kg BW	−33% CH_4_P/kg DMI	[68]

3-NOP = 3-nitrooxypropanol, HHC = halogenated hydrocarbons, CF = chloroform, BCM = bromochloromethane, DM = dry matter, DMI = dry matter intake, BW = body weight, CH_4_P = CH_4_ production, CH_4_Y = CH_4_ yield, N/A = Not available.

**Table 2:** Summary of the data on the effect of red seaweed used for enteric methane mitigation.

*Asparagopsis* Species	Animal Type	Dose	Effect on Emissions	Reference
*A. armata*	Dairy cattle	0.5% OM 1% OM	−26.4% CH_4_P −67.2% CH_4_P	[73]
*A. taxiformis*	Dairy cattle	0.25% DM 0.5% DM	No effect^1^ −34.4% CH_4_P	[74]
*A. armata*	Dairy cattle	134 g/d * 145 g/d *	−44% CH_4_P −39% CH_4_P	[75]
*A. taxiformis*	Beef cattle	0.25% OM 0.5% OM	−45% CH_4_Y −68% CH_4_Y	[76]
*A. taxiformis*	Beef cattle	0.05% OM 0.1% OM 0.2% OM	No effect −40% CH_4_P −98% CH_4_P	[77]
*A. taxiformis*	Sheep	0.5% OM 1% OM 1–1.5% OM 1.2–3.0% OM	No effect −52.2% CH_4_P −61.9% CH_4_P −81.3% CH_4_P	[78]

* = Treatments consisted of *A. taxiformis* steeped in canola oil without (134 g/d) and with (145 g/d) seaweed biomass, OM = organic matter, DM = dry matter, CH_4_P = CH_4_ production, CH_4_Y = CH_4_ yield.

**Table 3:** Summary of the data on the effect of whole plants or tannins, saponins, and essential oils used for enteric methane mitigation.

PSM Type	Plant Species or Specific PSM	Animal Type	Dose	Effect on Emissions	Reference
Tannins	HT	Beef cattle	6.5 g/kg DM 13 gm/kg DM 26 g/kg DM	−11.1% CH_4_Y −14.7% CH_4_Y −33.6% CH_4_Y	[99]
Tannins	HT and CT	Beef cattle	~14.6 g/kg DM	~−9.89% CH_4_P	[100]
Tannins, saponins	*Samanea saman*	Crossbred cattle	10% DM 20% DM 30% DM	−25.8% CH_4_P −40.39% CH_4_P −50.9% CH_4_P	[101]
Tannins	*Acacia saligna* *Leucaena leucocephala*	Sheep	50% DM 50% DM	−12.3% CH_4_P −10.5% CH_4_P	[102]
Saponins	Tea saponin	Sheep	5 g/kg DM 10 g/kg DM 20 g/kg DM	−28% CH_4_P −35.8% CH_4_P −47.9% CH_4_P	[108]
Saponins	*Enterolobium cyclocarpum*	Sheep	0.15 kg DM 0.3 kg DM 0.45 kg DM	−21.1% CH_4_P −36.6% CH_4_P −26.1% CH_4_P	[109]
Saponins	Tea saponin	Sheep	3 g/animal/day	−27.7% CH_4_P	[110]
Saponins	Tea saponin	Sheep	2 g/animal/day	−8.8% CH_4_P L/kg BW^0.75^	[111]
Saponins	*Medicago sativa* hay to provide saponin rates	Sheep	100 mg/kg BW 200 mg/kg BW 400 mg/kg BW	−5.7% CH_4_P −6.46% CH_4_P −22.62% CH_4_P	[112]
Saponins	*Medicago sativa*	Buffaloes	30% of diet	−21% CH_4_P	[113]
Saponins	Tea saponin	Beef cattle	20–30 g/animal/day	No effect	[116]
Saponins	Tea saponin	Dairy cattle	0.52% DM	No effect on CH_4_P +14% CH_4_Y	[117]
Saponins	Tea saponin	Dairy cattle	0.5% DM	No effect CH_4_P	[118]
Essential oils	Cinnamaldehyde, eugenol, carvacrol, capsicum oleoresin	Sheep	200 mg/kg DM 400 mg/kg DM	−27.7% CH_4_P kg DOM −35.9% CH_4_P kg DOM	[127]
Essential oils	Eucalyptus EO Anise EO	Sheep	0.5 g/animal/day 0.5 g/animal/day	No effect No effect	[129]
Essential oils	Ajwain, garlic, and cinnamon leaf EO	Buffaloes	0.15 mL/kg DMI 0.3 mL /kg DMI	−14.1% CH_4_P L/kg DMI −14.2% CH_4_P L/kg DMI	[130]
Essential oils	A blend of EO and pepper extract	Beef cattle	150 mg/kg DM	+11% CH_4_P +13.6 CH_4_Y	[131]
Essential oils	Thymol, eugenol, vanillin, limonese, guaiacol	Beef cattle	1 g/animal/day 2 g/animal/day	No effect No effect	[133]
Essential oils	Oregano EO	Dairy cattle	50 mg/kg DM	No effect	[134]
Essential oils	A blend of EO	Dairy cattle	1 g/animal/day	−8.8% CH_4_P	[135]

PSM = plant secondary metabolite, HT = hydrolyzable tannins, CT = condensed tannins, EO = essential oil, DM = dry matter, DMI = dry matter intake, BW = body weight, CH_4_P = CH_4_ production, CH_4_Y = CH_4_ yield.

**Table 4:** Summary of the data on the effect of nitrates and sulfates on enteric methane mitigation.

Substance Used	Animal Type	Dose	Effect on Emissions	Reference
Nitrate	Dairy cattle, beef cattle, and sheep	17.2–22.2 g/kg DM	−10–22.1% CH_4_Y	[61]
Nitrate	Sheep	2.6% DM	−32% CH_4_P	[161]
Sulfate		2.6% DM	−16% CH_4_P	
Nitrate + sulfate		2.6% DM each	−47% CH_4_P	
Nitrate	Dairy cattle	~16.7 g/kg DM	−20.4% CH_4_P	[164]
	Beef cattle		−10.1% CH_4_P	

DM = dry matter, CH_4_Y = CH_4_ yield, CH_4_P = CH_4_ production.

**Table 5:** Summary of the data on the effect of probiotics on enteric methane mitigation.

Microorganism Used	Animal Type	Dose	Effect on Emissions	Reference
*Lactobacillus pentosus* D31		6 × 10^10^ CFU/animal/day	−13% CH_4_P	
*Lactobacillus bulgaricus*	Sheep	3 × 10^10^ CFU/animal/day	No effect	[174]
*Propionibacterium freudenreichii* 53-W		6 × 10^10^ CFU/animal/day	+16% CH_4_P	
*Propionibacterium freudenreichii* 53-W	Dairy cattle	2.9 × 10^10^ CFU/animal/day	No effect	[175]
*Lactobacillus pentosus* D31		3.6 × 10^10^ CFU/animal/day	No effect	
*Lactobacillus bulgaricus*		4.6 × 10^10^ CFU/animal/day	No effect	
	Sheep	2.5 × 10^8^ CFU/animal/day	−12% CH_4_P DDMI	[179]
*Bacillus licheniformis*		2.5 × 10^9^ CFU/animal/day	−12% CH_4_P DDMI	
		2.5 × 10^10^ CFU/animal/day	−9.3% CH_4_P DDMI	

CFU = colony-forming units, CH_4_P = CH_4_ production, CH_4_P DDMI = CH_4_ production per digestible dry matter intake.

## Data Availability

Data sharing not applicable.
